# Prodromal multiple sclerosis: considerations and future utility

**DOI:** 10.1007/s00415-023-12173-4

**Published:** 2024-02-11

**Authors:** Katharine E. Harding, Karim L. Kreft, Yoav Ben-Shlomo, Neil P. Robertson

**Affiliations:** 1 Department of Neurology, Aneurin Bevan University Health Board, Royal Gwent Hospital, Cardiff Road, Newport, NP20 2UB UK; 2 Department of Neurology, Cardiff and Vale University Health Board, University Hospital of Wales, Heath Park, Cardiff, CF14 4XN UK; 3 Bristol Medical School, Population Health Sciences, Bristol, BS8 2PS UK; 4 Division of Psychological Medicine and Clinical Neuroscience, Department of Neurology, Cardiff University, University Hospital of Wales, Heath Park, Cardiff, CF14 4XN UK

**Keywords:** Multiple sclerosis, Prodrome

## Abstract

A multiple sclerosis (MS) prodrome has recently been described and is characterised by increased rates of healthcare utilisation and an excess frequency of fatigue, bladder problems, sensory symptoms and pain, in the years leading up to clinical onset of disease. This important observation may have several potential applications including in the identification of risk factors for disease, the potential to delay or prevent disease onset and early opportunities to alter disease course. It may also offer possibilities for the use of risk stratification algorithms and effective population screening. If standardised, clearly defined and disease specific, an MS prodrome is also likely to have a profound influence on research and clinical trials directed at the earliest stages of disease. In order to achieve these goals, it is essential to consider experience already gleaned from other disorders. More specifically, in some chronic neurological disorders the understanding of disease pro-drome is now well advanced and has been successfully applied. However, understanding of the MS prodrome remains at an early stage with key questions including the length of the prodrome, symptom specificity and potential benefits of early intervention as yet unanswered. In this review we will explore the evidence available to date and suggest future research strategies to address unanswered questions. In addition, whilst current understanding of the MS prodrome is not yet sufficient to justify changes in public health policy or MS management, we will consider the practical utility and future application of the MS prodrome in a wider health care setting.

## Introduction

A disease prodrome can be defined as an early, often non-specific, set of signs and/or symptoms that indicate onset of disease before typical signs and/or symptoms manifest to fulfil diagnostic criteria [1]. It is important to note that the prodrome is distinct from the “pre-clinical” phase of disease, where it may be possible to detect pathophysiological changes with the use of biomarkers, but for which there may be no clinical features apparent and individuals are unaware of any differences from their normal condition. In recent years, a prodrome has been identified in a number of neurological diseases including Parkinson’s disease (PD) [2], Alzheimer’s disease (AD) [3–6], and multiple sclerosis (MS) [7–16], providing some important insights into wider aspects of disease. In the case of MS, recent research has identified detectable differences in health-care utilisation and symptom frequency in the years leading up to a confirmed diagnosis [7–16]. These observations have in turn led to increased debate on whether and how this information might be applied to improve population health.

In theory, identification of a prodromal phase of disease could be valuable for several reasons. First, it might allow researchers to narrow down aetiological causes of disease and identify relevant risk factors. Second, defining risk factors that are active at key time points may offer opportunities to modify disease progression. Last, it offers the potential to enable earlier identification of disease and the potential for more timely treatment with the possible delay in disease onset and/or prevention of longer-term complications. However, before these lofty ideals can be achieved, it is worth pausing to consider the remaining obstacles to overcome before determining whether increased efforts to detect and define prodromal disease across diverse populations could eventually offer practical benefits for those individuals at risk of MS. In this Neurological Update we will discuss dilemmas surrounding the practical utility of the MS prodrome and areas for future research.

## Current evidence

Evidence for a prodrome in MS has been accumulating for some years (Table 1). Use of linked health administrative data in large populations has allowed the identification of patterns of increasing healthcare access in the years leading up to the first symptoms of MS [7–16]. Detailed analysis of these patterns has demonstrated an increasing frequency of outpatient encounters, hospital admissions and prescriptions for both physical symptoms such as pain, urinary tract problems, sensory symptoms and fatigue [7, 10, 11, 13], as well as neuropsychiatric symptoms including anxiety and depression [8, 10, 13]. It remains unclear to what degree these symptoms are related to MS pathophysiology, are MS risk factors or secondary to confounding variables that predict MS. In addition, the enhanced medical surveillance associated with vague symptoms may result in an association with MS due to “ascertainment bias”.

**Table 1:** Studies that have examined for prodromal features of multiple sclerosis

Author	Year	Country	Cases (n)	Controls (n)	Data source	Findings
Berger et al. [7]	2013	USA	5305	0	Medical insurance claims	1534 (28.9%) were diagnosed with fatigue prior to the index new MS diagnosis. Among the patients diagnosed with fatigue, 10.4% were also prescribed a fatigue-related medication. Fatigue was the earliest symptom in nearly 40% of patients. 30.8% of patients experienced only fatigue (and no other MS-related symptoms) in the three-year period prior to the index new MS diagnosis
Sinay et al. [17]	2015	Argentina	75	75	Clinical cohort linked to school records	There was no difference between cases and controls in rates of repeating a school year or dropping out of high school. Cases had lower marks in maths, language and overall average in the last three years of high school cycle compared to controls. This effect was largest for those with short time between finishing school and onset of MS. Similarly, no effect was seen for exam marks ≥ 3 years before the end of high school
Hoang et al. [8]	2016	Denmark	5084	24,771	Health administrative records	There was an increased risk of anxiety and depression (combined) in the two years before MS diagnosis [OR 1.40, 95% CI 1.05–1.88) but not significantly increased in the two years after diagnosis (OR 1.23, 0.92–1.64). OR was 1.4 (1.18–1.62) for depression in the MS population compared with controls, and the OR was 1.1 (0.78–1.43) for anxiety compared with controls
Wijnands et al. [9]	2017	Canada	14,428	72,059	Health administrative records, MS registry	More frequent hospital admissions, outpatient visits, and prescriptions for people who went on to develop MS compared to controls, detectable up to 5 years before MS onset
Disanto et al. [10]	2018	United Kingdom	10,204	39,448	Health administrative records	People who went on to develop MS had a higher frequency of physical (nausea and vomiting, urinary dysfunction, headache, eye pain, fatigue, back/neck pain) and psychiatric symptoms (anxiety, depression, insomnia and eating disorders) in the years leading up to MS onset. There was a positive association between the number of symptoms recorded and risk of future MS, with a 51%, 29%, and 20% increased risk of MS for each additional symptom at 0 to 2, 2 to 5, and 5 to 10 years before index date, respectively
Högg et al. [11]	2018	Canada	8669	40,867	Health administrative records	A physician encounter for a cerebrovascular, central or peripheral nervous system-related disease or disorder of the sense organs was associated with two to fivefold higher odds of MS
Marrie et al. [12]	2019	Canada	1155 CIS-MS cases, 20,638 CIS-non MS cases	108,726	Health administrative records	For both CIS-MS and CIS-non MS cases, there were higher rates of hospitalisation, physician visits and prescriptions than controls leading up to the CIS diagnosis. For CIS-non MS cases, this was detectable in all five years leading up to CIS diagnosis, and for CIS-MS cases was largely seen in the year before CIS diagnosis
Wijnands et al. [14]	2019	Canada	2058	9837	Health administrative records and MS registry	1887 R-MS cases, 171 PPMS cases. There was no difference in the number of physician encounters in the 5 years before MS onset. However, there was a difference in the outpatient specialties that patients visited: cases of PPMS had 92% more nervous system-related encounters and 71% fewer pregnancy/childbirth encounters relative to R-MS
Wijnands et al. [13]	2019	Canada	13,951	66,940	Health administrative records	There was an increase in number of physician encounters in the cases compared to controls during the five years leading up to first MS symptom. When classified by physician speciality there was a threefold to ninefold increases in visits to a neurologist (RR = 9.02, 95% CI: 7.53–10.80), neurosurgeon (RR = 3.63, 95% CI:2.81–4.69), or neurorehabilitation (RR = 3.20, 95% CI: 2.86–3.57), as well as increased number of encounters with a urologist (80%), ophthalmologist or otolaryngologist (76%), psychiatrist (66%) and internal medicine physician (53%)
Lebrun-Frénay et al. [19]	2020	Multi-country	451		Clinical RIS cohort	Mean age at RIS diagnosis was 37.2 years. Headache (42%) was the commonest reason for an initial MRI scan. The cumulative probability of a clinical event at 10 years was 51.2%. Of the 173 patients who developed MS symptoms, 21 (11.7%, 62% female) fulfilled criteria for primary progressive MS. In univariate analysis, factors associated with increased risk of a first clinical event were a younger age (than 37 years) at RIS diagnosis, positive CSF, and presence of either infratentorial or spinal cord lesions. Radiological activity during follow-up was also associated with increased risk of a clinical event (HR = 1.81 [95% CI 1.31–2.52], *p* < 0.001)
Yusuf et al. [15]	2021	Canada	6,863	31,865	Health administrative records and MS registry	This study investigated prevalence of pain, fatigue, sleep disorders and anaemia in the five years preceding MS onset compared to controls. Pain was the commonest symptom (50% in MS cases, 33% in controls), and all symptoms were more common in those who would go on to develop MS than controls (pain, odds ratio [OR] 2.15; sleep disorders OR 2.61; anaemia OR 1.53; fatigue OR 3.37)
Yusuf et al. [16]	2022	Canada	6863	31,865	Health administrative records	During the five years preceding a first demyelinating event, relative rates for males were 15% greater for total physician visits, and 21% higher for total hospital admissions, than relative rates for females. Age-specific effects included a 17% higher relative rate among older people with MS for visits to a GP, while younger people with MS had a 15–45% increased relative rate for ophthalmologist and sensory-related visits, and a 18% higher relative rate for cardiovascular-related drug prescription

Studies from Canada [9, 11–13, 15, 16], the USA [7], and Denmark [8] have identified a detectable increase in secondary healthcare use (outpatient clinic encounters, hospital admissions, and drug prescriptions) compared to age- and sex-matched controls, which rises steadily over the five years preceding the first clinical neuroinflammatory event. Analysis of the reasons for these encounters has identified higher frequency of pain and sensory symptoms [10, 11, 13, 15], urinary tract involvement [10, 13], and anxiety and depression [8, 10, 13]. There are also increased attendances at neurology, neurosurgery, neurorehabilitation, urology, ophthalmology, ENT, psychiatry and internal medicine clinics. A similar pattern was seen in a UK study of primary care data, with increased attendances for pain, urinary dysfunction, fatigue, anxiety and depression [10].

Within these data, sex- and age-specific effects have also been identified. While there is a higher rate of clinic attendances and hospital admissions for all those later diagnosed with MS compared to the general population, there is a significantly higher rate of clinic attendances and hospital admissions in males (adjusted rate ratio, aRR, 1.67 and 1.73) compared to females (aRR: 1.45 and 1.43) [16]. Furthermore, people aged ≥ 50 who go on to develop MS have a 17% higher relative rate of GP visits, whilst younger people (aged < 50) have 15–45% increased relative rate of ophthalmology and sensory-related clinic visits [16]. Only a few studies have examined differences in prodrome between primary progressive MS (PPMS) and relapsing-onset MS (R-MS). One Canadian study found no difference in the number of encounters, but a difference in physician speciality. People with PPMS had 92% more nervous system-related clinical encounters and a 71% lower chance of obstetric/pregnancy-related encounters than people with R-MS [14]. This is likely to relate to the younger age and female preponderance of people with R-MS compared to PPMS.

## Prodromal window

As part of our understanding of the MS prodrome, and interpretation of available data, it is essential to understand the potential interval between onset of prodromal symptoms and the onset of definite MS symptoms. This will not only guide future studies but also allow the earliest possible identification of those individuals at risk. Most studies of the MS prodrome to date have originated from Canada and utilise a common approach to analysis, starting from five years before MS onset [9, 11–16]. These studies found detectable differences between controls and people who go on to develop MS even at the earliest point of analysis, although differences become more marked the closer the temporal period before clinical onset. One study from the UK was able to detect differences in symptoms recorded by GPs up to 10 years before MS symptom onset [10]. In addition, a study of school achievement detected differences in school test results during the last 3 years of school in individuals with a mean age of MS onset of 31 years [17], suggesting the MS prodrome may extend even further than 10 years before clinical onset, though this is probably reflecting more of a pre-clinical than prodromal state.

Studies of radiologically isolated syndrome (RIS), which can be considered a pre-clinical phase of disease, may also be helpful in guiding estimates of prodrome duration. RIS is defined as the incidental identification of typical CNS white matter abnormalities on MRI in the absence of a history of relevant neurological dysfunction [18]. The increasing identification of RIS has paralleled the increasing availability of MRI with around half of those individuals identified with RIS having undergone such a scan for a headache indication. In addition, long-term follow-up of people with RIS demonstrated that at 10 years, 51.2% had developed at least one clinical event, and of those, 11.7% had symptoms consistent with PPMS [19]. The risk of a clinical event increased with younger age at RIS diagnosis, positive oligoclonal bands, presence of infratentorial lesions and/or presence of spinal cord lesions in adjusted models. This median time of 10 years to clinical symptom onset therefore suggests that the prodrome may be at least of this duration [19], albeit with differences between future MS cases and controls becoming more marked closer to clinical MS onset.

Finally, a few case series from the twentieth century have reported post-mortem pathological studies identifying demyelinating lesions in people not suspected to have MS during life [20]. However, there are limitations to these studies: it is not possible to know whether the individuals were truly asymptomatic and if they had a normal neurological examination, or whether they would have been diagnosed with MS using modern diagnostic criteria [21]. All of these data suggest that the scope of the prodromal window may be highly variable and problematic to define with accuracy.

## Lessons from other diseases

**Fig. 1:**
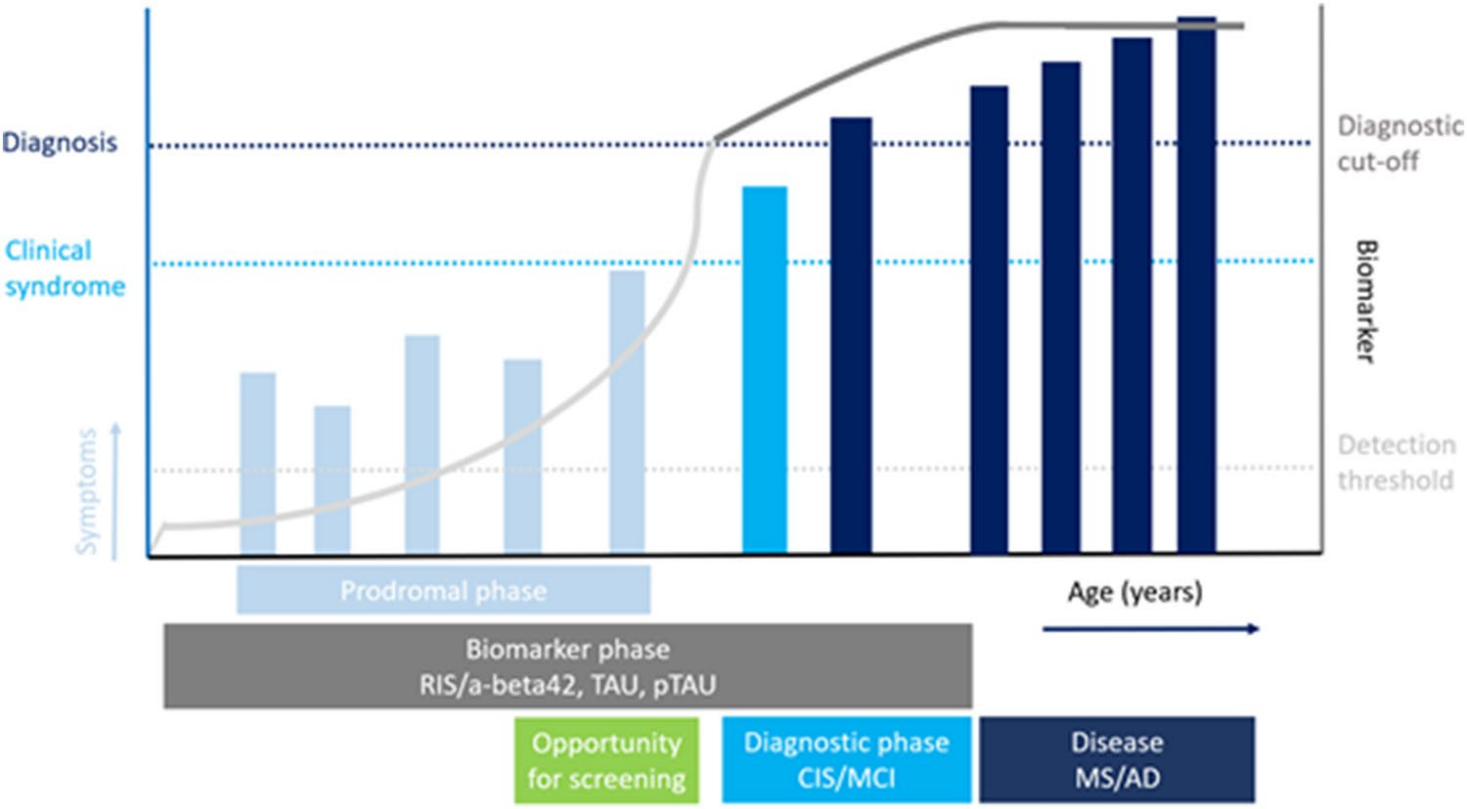
Prodromal symptoms in the development of MS and AD

Studies in neurological and non-neurological diseases to date have identified a range of relevant prodromal symptoms. In AD a similar pattern to MS is observed, although prodromal onset is later in life. Several years before onset of mild cognitive impairment (MCI), which might be considered comparable to the clinically isolated syndrome (CIS) in MS, behavioural and psychiatric symptoms occur in people who go on to develop AD [5, 6]. Raised levels of blood and CSF based biomarkers of AD pathology also occur several years before onset of MCI [3, 4] (Fig. 1). Lastly, in postmortem studies, AD pathology (amyloid depositions and neurofibrillary tangles) has been found in individuals without clinical signs of dementia, although these findings are less severe compared to people with a clinical diagnosis of AD [22] or PD [23].

The last decade has also seen a huge growth in research examining prodromal PD, identifying both nonmotor, mild motor features, neuroimaging and tissue markers that predict an increased risk of future clinical PD [24]. These predictors are seen in both population-based cohort studies as well as high risk populations such as REM sleep behaviour disorder (RBD), pure autonomic failure and those with a mutation in the GBA gene; one of the genetic risk factors for PD [25]. In 2015 the Movement Disorder Society (MDS) published an algorithm to determine the probability of prodromal PD using a Bayesian statistical approach [2]. The “prior probability” of prodromal PD is initially based on age and a series of variables are then added to produce a cumulative likelihood ratio and hence a posterior probability of prodromal PD. If this exceeds 80%, probable prodromal PD is diagnosed. Neuroimaging can also be helpful especially in patients with RBD and hyposmia where pathological DaT SPECT scans can indicate accelerated phenoconversion [26]. The prodromal algorithm has been validated across different datasets [27, 28] and uses two different classes of variables; (i) aetiological exposures such as pesticide use or genetic risk and (ii) prodromal features e.g. constipation, olfactory loss etc. as the tool is designed to deliver estimates of risk rather than providing a diagnosis. Differences in prodromal symptoms may also differ depending on whether pathology spreads in a top down (brain first) or bottom up (body first) fashion. Detailed imaging of patients with PD has identified a difference between those starting with RBD symptoms and those without RBD as a prodromal symptom. RBD is hypothesised to be a marker for body-first spread, with pathology beginning in the enteric or peripheral nervous system and subsequently spreading to the brain. This is supported by the observation that cardiac and colonic changes happen before brain changes using FDOPA-PET imaging can be identified. Conversely, those without RBD as a prodromal symptom are thought to have pathological changes first in the brain, and later spreading to the autonomic and enteric nervous systems. In these people, brain changes on FDOPA-PET are observed before cardiac or colonic changes [29].

The identification of the causative genetic mutation in Huntington’s disease (HD) has allowed the identification of pre-manifest carriers of the mutation. Longitudinal study of such individuals has identified a number of early symptoms, including autonomic symptoms [30] and neuropsychiatric symptoms including depression, apathy, irritability and altered executive function [31, 32]. Early investigation into biomarkers in HD suggests a promising role for serum neurofilament light protein; this has been shown to correlate with clinical and radiological changes in HD [33].

Prodromal symptoms have also been described in other auto-immune diseases. For example autoantibodies in rheumatoid arthritis (RA) are present before a clinical diagnosis of joint inflammation [34–36], as well as an increased prevalence of mental health problems [37, 38], and carpal tunnel syndrome [38]. In inflammatory bowel disease, mainly in Crohn’s and coeliac disease, prodromal symptoms have also been identified [39, 40] but not in ulcerative colitis [39, 40], (Table 2) although the number of participating patients is relatively smaller and those studies may be underpowered rather than reflecting differences in disease development.

**Table 2:** Prodromal and pre-clinical features in other neurological and auto-immune disorders

Disease	Prodromal symptoms	Pre-diagnostic symptoms	Biomarkers in prodromal phase	Pre-diagnostic disease
Multiple sclerosis [7–16]		–	Radiologically isolated syndrome (RIS) Oligoclonal bands	Clinically isolated syndrome (CIS)
Alzheimer’s disease [3–6]	Mild behavioural problems, psychiatric disorders	Mild cognitive symptoms	Aβ42, TAU, pTAU	Mild cognitive impairment (MCI)
Parkinson’s disease [24, 54]		REM sleep behavioural disorder (RBD), anosmia, non-motor features	DAT scan α-synuclein tissue deposition and seed amplification	Prodromal PD
Huntington’s disease [30–33]	Depression, apathy, irritability, executive dysfunction		CAG triplet repeat expansion in the *HTT* gene Serum neurofilament light	Peri-manifest HD
Rheumatoid Arthritis [30–34]	Arthralgia, fatigue, reduced mental health, cardiovascular diseases, carpal tunnel syndrome	Pain, stiffness swelling of the joint, joint tenderness, morning stiffness	Rheumatoid factor IgM, anti-CCP IgG	–
Crohn’s disease [35, 36]	Depression	Irritable bowel syndrome	–	–
Ulcerative colitis [35, 36]	–		–	–

## Methodological issues

The rationale for identification of prodromal disease outside of research is contingent on improving patient outcomes and is similar in principle, but is not the same as screening for disease. In the latter, subjects may be identified pre-clinically before any symptoms or signs of disease (e.g. breast mass only detectable on mammography). Currently prodromal disease is usually defined by the presence of some detectable feature albeit so mild (e.g. cognitive) as to not even necessitate seeking health care. As with screening, early treatment may appear effective, even if it is not, due to “lead time” bias [41], which simply brings forward the time of diagnosis making survival appear improved even if the natural history of the disease is not altered.

Any prodromal diagnostic tool will need to balance benefits with potential harms given the positive and negative predictive values of the diagnostic tool. Given the rarity of MS, even a tool with high specificity will still likely have a high ratio of false to true positive diagnoses, meaning that a large number of individuals who are detected by the instrument will turn out not to progress to clinical disease. It is likely that more expensive, invasive and definitive diagnostic tests will be required as a second stage. One obvious approach would be to stratify individuals into low, medium and high risk of conversion bands (based on empirical probabilities from cohort studies), thereby restricting further testing on some pragmatic threshold and avoiding “over-labelling”. The choice of a cut-point will obviously depend on cost-effectiveness, as well as other ethical considerations. In addition, any proposed diagnostic and/or prognostic tool will need to be validated both across and within populations. So for example, the prevalence of other conditions that might mimic the diagnostic exposures, and hence increase the false positive rate, will vary across High Income (HIC) and Low Middle Income countries (LMIC). Similarly, within a population the predictive value may differ by age group or gender depending on what symptoms/signs are used and hence these factors may need to be accounted for when deriving a prognostic probability. Ultimately the value of any prodromal tool is whether it correctly identifies individuals who may benefit from earlier diagnosis without resulting in over-diagnosis or other harm.

## Utility

Successful and widespread application of a tool to identify individuals in disease prodrome, outside of research, will require the availability of an effective intervention to delay, prevent or otherwise beneficially modify the natural history of disease. Whilst these exist for those with MS, it is currently lacking even for some earlier forms of disease i.e. CIS and RIS, although a range of clinical trials are currently attempting to provide such an evidence base [42–44]. Conversely, the identification of a prodromal phase with a high level of predictive value may also lead to substantial adverse psychosocial consequences which need to be considered. As a result, the level of acceptable post-test probability will need to be carefully balanced and risk acceptance may also change depending on the nature of available interventions.

**Fig. 2:**
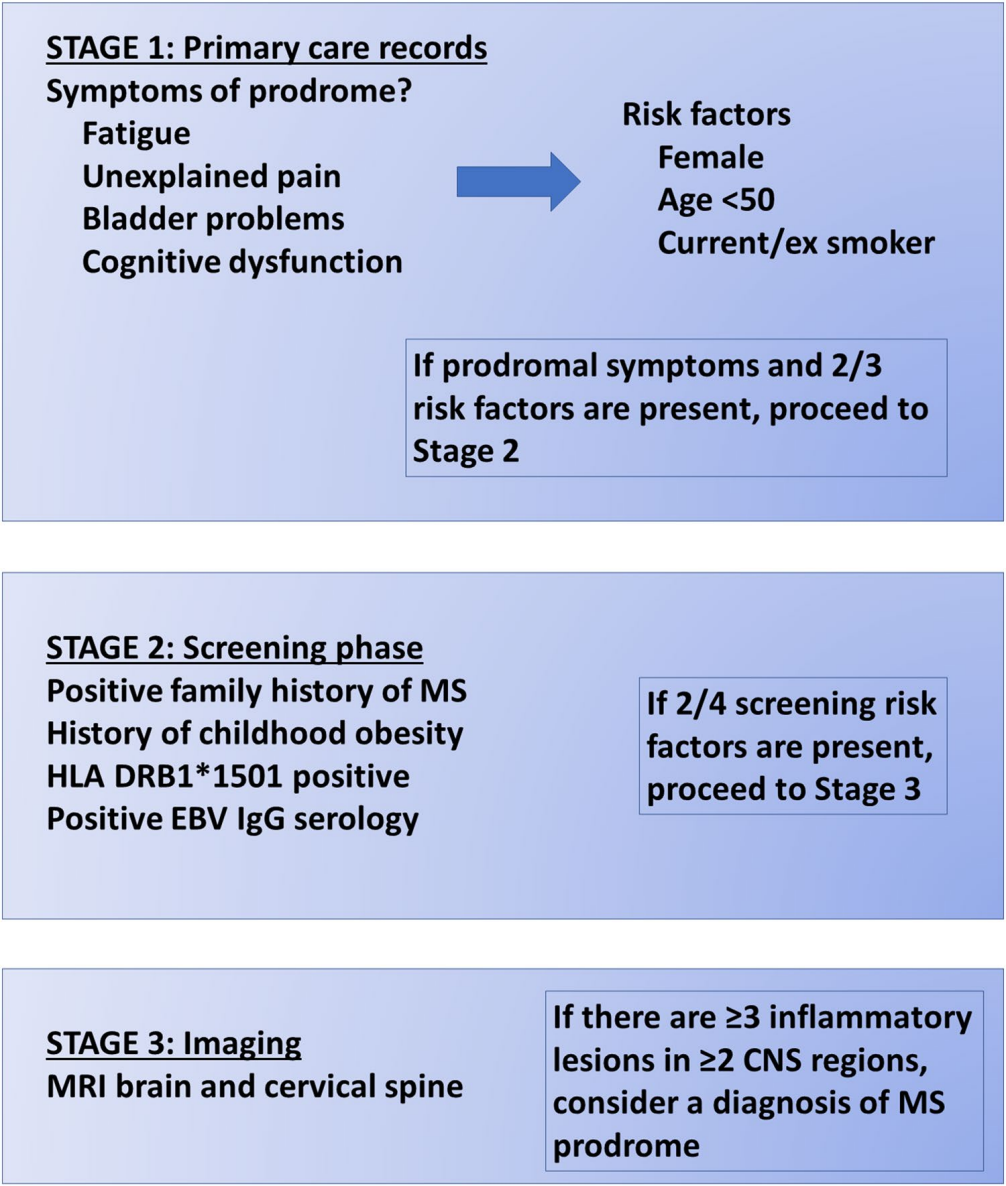
Proposed hypothetical algorithm for MS prodrome screening

Some of the characteristic features of MS relevant to analysis of prodromal features include a predominance of females, in an approximate ratio of 2–3:1, a broad age of onset (occurring in every decade of life but commonly in the third and fourth decades) and a heterogeneous natural disease course [45] with some benign phenotypes, which will provide challenges. Furthermore, the predictive value of a pre-diagnostic tool will need to be optimised by incorporating both prodromal features and established risk factors for MS. These include HLA DR15 status, 201 non-HLA SNPs that account for around 39% of MS heritability [46], a family history of MS with lifetime risk for monozygotic twins of between 25 and 45% and for siblings of 2–3%, childhood obesity, socioeconomic status, smoking, EBV serology and Vitamin D levels [47]. Figure 2 shows a putative hypothetical algorithm incorporating known risk factors that could potentially be used to screen for prodromal MS. Finally, the timing and frequency of data collection for interpretation will be key to success. At present the interval between the prodrome, biological and clinical onset remains unclear. Whilst the minority of patients present with a relevant isolated area of CNS inflammation with an appropriate clinical correlate, the majority have evidence of pre-existing T2 lesions on MRI. Although some radiological characteristics have been employed to approximate time from biological onset such as presence of T1 holes, burden of T2 lesions, focal or generalised atrophy and perilesional oedema, more specific estimates are elusive and current models are based on data from RIS cohorts and rate of accumulation of lesions in longitudinal MRI studies. A detailed understanding of how the prodromal period meshes with biological onset will need to be developed from long-term studies of high-risk groups such as RIS.

The window between prodrome, biological and clinical onset will be key in determining the appropriate timing or age of application of a prognostic tool to identify and test any at-risk population. Any tool will need to employ widely available tests or processes. These may include self-completion questionnaires, data linkage, electronic records, or blood tests, with other tests such as MR imaging and CSF examination being limited to use at a second stage only where the prior probability is already elevated. More recent reports of MS prodrome have tended to use reporting of common symptoms and drug usage. However, the utility of this methodology will necessitate reliable reporting and recording of symptoms, appropriate infrastructure, safety of process and confidentiality, high levels of electronic patient record (EPR) coverage, access to data and analysis of large datasets delivered at a reasonable cost. Whilst initial reports of the MS prodrome have stimulated interest and conversation, more widespread application of any prognostic tool requires evidence for reproducibility in different populations with high levels of sensitivity and specificity. In addition, the consequences (both in terms of costs as well as logistics) for health care providers on managing the identification of at-risk populations who are likely to require further clinical and radiological assessment will need to be carefully considered.

## Areas for further research and development

If prodromal symptoms are able to reliably identify people at high risk of developing MS by using linkage of data and automated data analysis (e.g. machine learning (ML) approaches), several elements will need to be taken into consideration. Firstly, the value of any predictive model will depend on the quality of available data. Secondly, the diagnostic accuracy will need to be compared with conventional statistical models as these may or may not be superior [48] and overinterpretation of ML models may result in models of very limited generalizability resulting in poor prediction and distrust [49]. Any model developed in one health care system will need replication and validation to other regions and countries due to lack of standardisation of data collection and cultural differences in health care seeking behaviours. Thirdly, important aspects of data confidentiality need to be clarified, especially if data sources are to be linked across different organisations (e.g. primary and secondary care) to enhance utility.

It is worth noting that several prodromal symptoms overlap between different diseases (Table 2). Future evaluation of a prodromal model for MS should not only be compared with healthy controls, but also with other diseases with overlapping symptoms. For example, depression is a symptom which has been identified as a prodromal feature of MS [8, 10, 13], but is also associated with RA [37], AD [6], and PD [2], which may limit its use as a feature of the prodrome to predict MS susceptibility. This highlights the need for an algorithmic approach to diagnosing prodromal MS, taking a number of different factors into account in order to risk-stratify patients, similar to the MDS research criteria for prodromal PD [2].

If we are able to overcome these important aspects in the development of a reliable prediction model based on prodromal features, the next step will be to use this model to plan clinical trials to investigate whether the onset of MS can be prevented or delayed. This could explore pharmacological interventions as well as conservative measures such as lifestyle changes including exercise. Similar neuroprotective trials are already beginning in PD [50], AD [51], and HD [52].

Qualitative research on the acceptability of diagnosing prodromal MS for patients and their family will also be important. Specifically, whether individuals would be prepared to undergo testing, what barriers there might be to participation, and whether the burden and efficacy of potential treatments would affect willingness to participate. For example, the risks of taking vitamin D would be quite different to those of an immunosuppressive monoclonal antibody. The ARISE trial, which compared dimethyl fumarate to placebo in RIS, had significant difficulty recruiting patients, necessitating early termination of the study and modification of the statistical analysis [44]. Whether one was to consider population-based screening for prodromal MS or only focussing on high-risk populations, an economic justification will be necessary weighing up the necessary financial resources compared to any benefit in terms of quality of life adjusted life years.

Finally, further epidemiological studies will be of value if prodromal MS can be reliably identified. The cascade of events leading up to MS onset, the influence of disease risk factors and the interplay between all of these factors are not clearly understood. Identification of a prodromal phase of MS would allow researchers to focus on the earliest stages of MS, which would lessen the impact of confounding factors or reverse causation and allow investigation into the underlying mechanisms that lead to MS. This may provide new insights into putative treatments for neuroprotection and prevention of neuroinflammation.

## Implications for patient care

Although stratifying risk and pre-emptive interventions may appear attractive to policy makers and clinical services, the effect on individuals and their families will need to be carefully considered. As we have learnt from predictive testing in genetic disorders, the willingness to acquire this information varies depending on several factors including prior knowledge of the disorder (i.e., family history), disease severity, the predictive value of testing, individual interpretation of risk and the ability to alter the disease course [53]. Even the availability and knowledge of predictive testing may result in adverse effects including health anxiety, as well as having consequences for those identified as having high risk of disease, such as health and other insurance. However, to be effective, the application of prodromal analysis will need to be encompassed within health care policies. As well as resources for testing populations and the facilities for doing this, systems would need to be developed to manage those identified as positive from the algorithms within a comprehensive health strategy. The additional clinical resources needed to assess people for prodromal status will also affect other services, for example increased use of MRI scanning.

A key issue will be to continually reassess the value of identifying prodromal characteristics on an individual level in the context of available interventions. There will need to be clear benefit from early intervention either in terms of behavioural change or via timely application of therapies. Currently the evidence for this is absent but may change as randomised clinical trials increasingly focus on the very earliest phases of disease and effective neuroprotective and neuroreparative treatments start to emerge.

## Conclusions

The existing evidence base suggests that it may be possible to identify prodromal MS using existing data sources. However, it is clear that more research is required to develop optimal data collection methods (passive and/or active) and the algorithms to classify individuals that are likely to require further testing. If justified, this will necessitate large scale multi-country prognostic cohort studies that risk stratify community samples and cross-validate estimated probabilities both within and between countries. Such prognostic tools, however, currently remain at the level of research interest, though if developed would enable recruitment for future secondary prevention randomised controlled trials. These will require careful evaluation including measuring potential adverse effects both from labelling false positives as well as over-treatment for individuals who may have a more benign disease course. Demonstrating the cost-effectiveness of any prodromal detection and treatment programme will be essential before any policy changes can be recommended.
